# Targeted online health information was associated with more severe temporomandibular disorders

**DOI:** 10.22514/jofph.2025.081

**Published:** 2025-12-12

**Authors:** Yijun Li, Li Zhang, Zhiwei Cao, Yanyu Sun, Ziang Xu, Carolina Marpaung, Jun Wang, Xin Xiong

**Affiliations:** ^1^State Key Laboratory of Oral Diseases & National Center for Stomatology & National Clinical Research Center for Oral Diseases, West China Hospital of Stomatology, Sichuan University, 610041 Chengdu, Sichuan, China; ^2^School of Stomatology, Tianjin Medical University, 300070 Tianjin, China; ^3^Department of Prosthodontics, Faculty of Dentistry, Universitas Trisakti, 11440 Jakarta, Indonesia

**Keywords:** Temporomandibular disorders, Online healthy information, Somatic symptoms, Anxiety, Cyberchondria, Chain mediation

## Abstract

**Background**: Temporomandibular disorders (TMDs) are typical biopsychosocial conditions often accompanied by anxiety, somatization and even cyberchondria. Targeted online health information is increasingly prominent in digital era yet its psychological impact on TMDs remains underexplored. To examine the association between targeted online health information and TMDs severity, and to explore whether anxiety, cyberchondria and somatization mediate this relationship. **Methods**: Participants were evaluated using questionnaires including the five TMDs symptoms (TMDs-5T) scale, 7-item Generalised Anxiety Disorder scale (GAD-7), short-form version of the cyberchondria severity scale (CSS-12) and somatic symptom scale-8 (SSS-8). Data on targeted online health information, search frequency and perceived impact were also collected. Analyses were conducted using SPSS and Mplus. **Results**: A total of 588 valid responses were analyzed. Greater TMDs severity was significantly associated with targeted online health information delivery (β = 1.373, *p* < 0.001), anxiety (β = 1.714, *p* < 0.001) and cyberchondria (β = 1.641, *p* < 0.001). The chain mediation model revealed that both the total effect and direct effect (Targeted online health information → TMDs-5T) were significant (β = 2.261, *p* < 0.001; β = 1.003, *p* < 0.001). A significant indirect pathway was also identified, in which targeted online health information influenced TMD severity through somatization, anxiety, and cyberchondria (β = 0.210, *p* = 0.026). **Conclusions**: Exposure to targeted online health information was associated with greater TMD symptom severity, mediated by psychological factors such as somatization, anxiety, and cyberchondria. These findings underscore the importance of algorithmic ethics, policy oversight, and user education to mitigate psychological risks in digital health environments.

## 1. Introduction

Temporomandibular disorders (TMDs) are the second most common musculoskeletal condition after low back pain [1]. Approximately 27%–33% of the Chinese population have signs of TMDs [1, 2]. TMDs primarily affect the temporomandibular joint (TMJ), masticatory muscles and associated anatomical structures, and might lead to persistent pain and functional limitations, thereby significantly impairing the quality of life in affected individuals [3, 4, 5]. Although many cases show a fluctuating or recurrent course, epidemiological data indicate that approximately 15% of patients who seek clinical care progress to chronic TMD pain often persisting for 3 to 6 months or longer. This chronic subgroup is associated with sustained disability and higher healthcare demand [6, 7]. The etiology of TMDs is multifactorial, and the “biopsychosocial model” has been widely accepted as a comprehensive framework for understanding their pathogenesis [8, 9]. Previous studies have found that measures of psychological functioning can predict the first onset of TMDs [4, 10], while psychological factors including anxiety, somatization and stress reactions play a critical role in the development and progression of TMDs [11, 12, 13, 14, 15].

With the rapid development of online medical information sources and the growing public demand for active participation in health management and the reduction of healthcare costs, an increasing number of individuals are turning to the internet to obtain health-related information, including disease characteristics, symptom presentations and treatment recommendations [16, 17, 18]. Driven by this trend, the integration of big data analytics and user behavior tracking technologies has enabled digital platforms to deliver disease-specific content with high precision based on users’ search histories, health interests and demographic profiles [19]. Although such targeted delivery of online health information enhances content relevance, it may also amplify patients’ vigilance toward bodily symptoms and increase health-related concerns, thereby reinforcing levels of anxiety, somatic symptoms and tendencies toward cyberchondria [20, 21]. Cyberchondria refers to an excessive and often escalating preoccupation with one’s health, characterized by compulsive online searches for medical information, which may range from mild concern to severe health-related anxiety [22]. Not all online health information is equal. Reliable sources, such as professional websites or peer-reviewed publications, generally support informed self-management, while unverified sources, like forums or social media, may increase anxiety, somatization, and maladaptive behaviors [23]. Among individuals with heightened sensitivity, frequent exposure to vast amounts of unscreened or medically uncontextualized health information may lead to cognitive distortions and maladaptive health behaviors, such as repeated medical consultations, self-diagnosis, or self-modification of treatment regimens [24], which may aggravate the severity of TMDs symptoms [25]. It is therefore plausible that personalized online health information, while medically relevant, may indirectly aggravate TMD symptoms by activating these underlying psychological pathways.

As a result, investigating how targeted online health information influences the clinical manifestations of TMDs through psychological mechanisms is of notable importance. Such exploration contributes to a deeper understanding of behavioral response patterns in patients with TMDs and provides valuable insight for developing more tailored and effective intervention strategies. In this context, targeted online health information delivery may present a double-edged effect, offering potential benefits while simultaneously posing psychological risks.

The present study aimed to examine the relationship between targeted online health information delivery and the severity of TMDs. Furthermore, the study explored whether anxiety, cyberchondria and somatization function as mediating factors in this association. The null hypotheses were as follows: first, there would be no significant association between the severity of TMDs and the receipt of targeted online health information. Second, anxiety, cyberchondria and somatic symptoms would not serve as mediators in the relationship between targeted online health information and the severity of TMDs.

## 2. Materials and methods

### 2.1 Study design

To guarantee the precision and transparency of observational research, this study was carried out in compliance with the guidelines specified in the Strengthening the Reporting of Observational Studies in Epidemiology (STROBE) statement [26]. All experimental procedures were strictly in line with the ethical principles laid down in the Declaration of Helsinki and obtained approval from the Institutional Review Board of West China Hospital of Stomatology, Sichuan University (Approval Number: WCHSIRB-D-2024-464).​

This cross-sectional survey was conducted between August 2024 and November 2024 among community residents in Chengdu, China. Participants were required to complete an electronic questionnaire consisting of three main sections. In the first section, demographic information including age, sex, and educational level was obtained. In the second section, participants were asked to report whether they had received targeted online health information within the past two weeks, how frequently they searched for health-related information online, and how they perceived the impact of such information. The final section comprised four standardized questionnaires: the five TMDs symptoms (TMDs-5T) scale, the somatic symptom scale-8 (SSS-8), the 7-item Generalised Anxiety Disorder Questionnaire (GAD-7) and the short-form version of the cyberchondria severity scale (CSS-12). All four scales have been translated and validated for use in Chinese populations, demonstrating good reliability and validity [27, 28, 29, 30].

The inclusion criteria specified participants who were between 18 and 50 years of age, those who voluntarily consented to participate in the study, and those who were able to independently complete the questionnaire. Participants with a history of major psychiatric, autoimmune, cardiovascular, or endocrine disease were excluded. We screened responses for quality and excluded questionnaires that were factually inconsistent or irrelevant, contained personally identifying information, or showed identical extreme responses across all items. The required sample size was calculated using G*Power (version 3.1.9.3, G*Power Team, Düsseldorf, NRW, Germany). Based on a medium effect size (Cohen’s *f*^2^ = 0.15), a significance level of 0.05, and a power of 0.95, the minimum sample size was determined to be 326 participants. A total of 645 volunteers eventually participated in the study, exceeding the minimum requirement. Informed consent was obtained from all participants prior to their inclusion in the survey.

### 2.2 Measurements

Targeted online health information delivery was defined as receiving health-related content tailored to the individual within the past two weeks via commonly used social media platforms such as TikTok, Rednote, Weibo and Wechat. Frequency of online health information searching was assessed on a five-point scale, including: rarely, several times per year, several times per month, several times per week, and several times per day. For statistical analysis, “rarely” and “several times per year” were categorized as low frequency, whereas “several times per month”, “several times per week”, and “several times per day” were classified as high frequency. In addition, participants were asked to evaluate the perceived impact of online health information as positive, neutral, or negative.

#### 2.2.1 TMDs-5T

The TMDs-5T scale was used to identify participants with TMDs, as it has demonstrated high diagnostic accuracy in detecting TMDs [31, 32]. Each item is rated on a 4-point Likert scale: 0 = “not at all”, 1 = “yes, but not bothered at all”, 2 = “yes, bothered a little”, and 3 = “Yes, felt bothered a lot”. The total score ranges from 0 to 15, with higher scores indicating greater symptom severity. Based on the total score, TMD severity was categorized as follows: 0 = no TMDs, 1–5 = mild TMDs, 6–10 = moderate TMDs, and 11–15 = severe TMDs.

#### 2.2.2 GAD-7, SSS-8 and CSS-12

As in our previous study, the GAD-7, SSS-8, and CSS-12 were used to assess anxiety, somatic symptoms, and cyberchondria, respectively. The GAD-7 includes seven items scored from 0 to 4, with total scores ≥9 indicating anxiety. The SSS-8 comprises eight items rated from 0 to 4, with scores ≥9 suggesting significant somatic symptom burden. The CSS-12 consists of 12 items rated on a 5-point Likert scale, and a total score ≥36 reflects a high level of cyberchondria.

### 2.3 Statistical analysis

Statistical analysis was executed using SPSS software (version 21.0, IBM Corporation, Armonk, NY, USA), R (version 4.4.2), with a significance level set at 0.05. Mplus (version 8.3, Muthén & Muthén, Los Angeles, CA, USA) was used to conduct chain mediation. Quantitative data were depicted as mean ± standard deviation, while categorical data were presented in the form of frequencies and percentages. Continuous variables were reported as mean or median accompanied by standard deviation or interquartile range. Normality was examined using the Shapiro-Wilk test, and all continuous variables demonstrated significant deviation from normal distribution (*p* < 0.001). Therefore, non-parametric tests were adopted. Due to non-normal distribution, the Kruskal-Wallis test was used for group comparisons in age, education, GAD-7, SSS-8, CSS-12, and effect sizes (η^2^) were calculated to assess the magnitude of differences, interpreted as small (0.01), medium (0.06) and large (0.14) [33]. For the categorical variable of targeted online health information, group comparisons were assessed using the Chi-square test, and Cramer’s *V* was calculated to determine the effect size. Effect size thresholds were interpreted as small (*V* = 0.10), medium (*V* = 0.30), and large (*V* = 0.50), in accordance with Cohen’s criteria. Spearman correlation coefficients were calculated for TMDs-5T, GAD-7, SSS-8, and CSS-12, and Holm-Bonferroni correction was applied for multiple testing [34]. Correlation strength was interpreted using *r* values. The correlation coefficients were categorized as weak (0.10–0.29), moderate (0.30–0.49), or strong (0.60–1.00). Confidence intervals for effect sizes and mediation pathways were estimated using bias-corrected bootstrap procedures with 1000 resamples.

## 3. Results

Of the 645 distributed questionnaires, 588 valid responses (91.2%) were retained for final analysis after excluding incomplete or noncompliant entries. Within the overall sample, 297 individuals (50.5%) did not report TMDs, whereas 291 participants (49.5%) had TMDs-consistent symptoms per the TMDs-5T screener. Among the TMDs, 196 individuals were classified as mild TMDs (33.3%), 74 individuals as moderate TMDs (12.6%), and 21 individuals as severe TMDs (3.6%). The sex distribution consisted of 333 females (56.6%) and 255 males (43.4%). The results also indicated that the proportion of females in the TMDs group was significantly higher than that in the no TMDs group (*p* = 0.025). Additionally, the study revealed a significant association between TMDs severity and educational level (*p* < 0.001) (Table 1).

**Table 1.** Demographic data of the participants.

Demographics		No TMDs	Mild TMDs	Moderate TMDs	Severe TMDs	*p*-value
n (%)		297 (50.5%)	196 (33.3%)	74 (12.6%)	21 (3.6%)	Not applicable
Age (yr)						
	Mean ± SD	32.80 ± 13.00	29.00 ± 9.50	27.80 ± 8.50	29.70 ± 8.80	0.283
	Median	16.00	18.00	15.00	21.00	
Sex, n (%)						
	Female	150 (50.5%)	122 (62.2%)	48 (64.9%)	13 (61.9%)	0.025*
	Male	147 (49.5%)	74 (37.8%)	26 (35.1%)	8 (38.1%)	
Education level, n (%)						
	Secondary school and below	42 (14.1%)	50 (25.5%)	26 (35.1%)	5 (23.8%)	<0.001*
	Undergraduate or junior college	190 (64.0%)	94 (48.0%)	34 (45.9%)	11 (52.4%)	
	Postgraduate and above	65 (21.9%)	52 (26.5%)	14 (19.0%)	5 (23.8%)	

*: indicates significant differences between groups (p < 0.05). TMDs: Temporomandibular disorders; SD: standard deviation.

In Table 2, the proportion of individuals receiving targeted online health information is significantly higher in those with more severe TMDs (*p* < 0.001). Similarly, the frequency of online health information searching increased significantly with increasing TMD severity (*p* < 0.001). Scores on the GAD-7, SSS-8 and CSS-12 exhibit significant differences and increased significantly in proportion with the aggravation of TMD severity (*p* < 0.001). Although the difference was not statistically significant, a higher proportion of participants in the severe TMDs group perceive the impact of online health information as negative compared to those in the mild and moderate groups (*p* = 0.052).

**Table 2.** Differences in targeted online healthy information, frequency of online health information searching, perceived impact of online health information, GAD-7, SSS-8 and CSS-12 for different severity of TMDs.

Demographics		No TMDs	Mild TMDs	Moderate TMDs	Severe TMDs	*p*-value
Targeted online health information, n (%)						
	Yes	86 (29.0%)	83 (42.3%)	51 (68.9%)	20 (95.2%)	<0.001*
	No	211 (71.0%)	113 (57.7%)	23 (31.1%)	1 (4.8%)	
Frequency of online health information searching, n (%)						
	Multiple times per year and less	236 (79.5%)	140 (71.4%)	37 (50.0%)	8 (38.1%)	<0.001*
	Multiple times per month and above	61 (20.5%)	56 (28.6%)	37 (50.0%)	13 (61.9%)	
Perceived impact of online health information, n (%)						
	Positive	90 (30.3%)	55 (28.1%)	18 (24.3%)	8 (38.1%)	0.052
	Neutral	187 (63.0%)	123 (62.8%)	43 (58.1%)	9 (42.9%)	
	Negative	20 (6.7%)	18 (9.1%)	13 (17.6%)	4 (19.0%)	
GAD-7						
	Without	281 (94.6%)	148 (75.5%)	42 (56.8%)	6 (28.6%)	<0.001*
	With	16 (5.4%)	48 (24.5%)	32 (43.2%)	15 (71.4%)	
	Median (Q1–Q3)	1.0 (0.00–4.00)	4.0 (2.00–8.00)	7.0 (4.00–11.00)	14.0 (8.00–17.00)	
SSS-8						
	Without	253 (85.2%)	114 (58.2%)	27 (36.5%)	3 (14.3%)	<0.001*
	With	44 (14.8%)	82 (41.8%)	47 (63.5%)	18 (85.7%)	
	Median (Q1–Q3)	3.0 (1.00–6.00)	7.5 (4.00–11.00)	11.5 (8.00–18.75)	21.0 (14.00–25.00)	
CSS-12						
	Without	277 (93.3%)	174 (88.8%)	59 (79.7%)	6 (28.6%)	<0.001*
	With	20 (6.7%)	22 (11.2%)	15 (20.3%)	15 (71.4%)	
	Median (Q1–Q3)	20.0 (13.00–25.00)	25.0 (19.00–31.00)	30.0 (23.00–34.00)	39.0 (33.00–42.00)	

*: indicates significant differences between groups (p < 0.05). GAD: generalised anxiety disorder; SSS: somatic symptom scale; CSS: cyberchondria severity scale; TMDs: Temporomandibular disorders.

Fig. 1 illustrates the spearman correlation coefficients among the variables. In Table 3, effect size analysis (η^2^) was conducted to assess the magnitude of group differences. According to established thresholds, only psychosomatic variables including TMDs-5T (η^2^ = 0.958), SSS-8 (η^2^ = 0.275), GAD-7 (η^2^ = 0.261) and CSS-12 (η^2^ = 0.148) demonstrated large effect sizes. In contrast, demographic variables such as age (η^2^ = 0.010) and education level (η^2^ = 0.008) showed only negligible to small effects, indicating limited explanatory value. As shown in Table 4, TMD scores exhibit positive correlations with the presence of anxiety (β = 1.714, 95% confidence interval (CI) 1.032–2.396), somatization (β = 1.553, 95% CI 0.997–2.109) and cyberchondria (β = 1.641, 95% CI 0.916–2.367). Additionally, targeted online health information delivery emerges as a positive predictor of TMD scores (β = 1.373, 95% CI 0.895–1.85).

**Fig. 1.** The heatmap of the Spearman’s *rho* correlations among TMDs-5T, GAD-7, SSS-8, CSS-12 and clinical characteristics of all participants (Holm-Bonferroni adjusted). GAD: generalised anxiety disorder; SSS: somatic symptom scale; CSS: cyberchondria severity scale; TMDs-5T: five temporomandibular disorders symptoms. ***p value < 0.001.

**Table 3.** The effect sizes (η^2^ or *V*) of variables.

Variable	η^2^/*V*	LLCI	ULCI	Magnitude
Age	0.010	−0.003	0.030	Small
Edu	0.008	−0.005	0.030	Small
TMDs-5T	0.958	0.940	0.970	Large
SSS-8	0.275	0.210	0.330	Large
GAD-7	0.261	0.190	0.320	Large
CSS-12	0.148	0.090	0.200	Large
Targeted online health information	0.339	0.257	0.399	Median

*GAD: generalised anxiety disorder; SSS: somatic symptom scale; CSS: cyberchondria severity scale; TMDs-5T: five temporomandibular disorders symptoms; Edu: Education; LLCI: lower limit of confidence interval; ULCI: upper limit of confidence interval.*

**Table 4.** Univariate and Multivariate regression analysis of variables associated with scores of TMDs.

Variables		Univariate regression			Multivariate regression		
		β	*p*-value	95% CI	β	*p*-value	95% CI
Targeted online health information (Ref. No)							
	Yes	2.261	<0.001*	1.753–2.769	1.373	<0.001*	0.895–1.850
GAD-7 (Ref. without)							
	With	3.445	<0.001*	2.826–4.063	1.714	<0.001*	1.032–2.396
SSS-8 (Ref. without)							
	With	2.814	<0.001*	2.295–3.333	1.553	<0.001*	0.997–2.109
CSS-12 (Ref. without)							
	With	3.152	<0.001*	2.383–3.920	1.641	<0.001*	0.916–2.367

*: indicates significant differences between groups (p < 0.05). GAD: generalised anxiety disorder; SSS: somatic symptom scale; CSS: cyberchondria severity scale; CI: confidence interval; Ref.: reference.

Table 5 and Fig. 2 demonstrate that targeted online health information exerts a significant influence on TMDs-5T scores through multiple mediation pathways. The direct effect of targeted online health information on TMDs scores was significant (β = 1.003, 95% CI 0.538–1.467), indicating that after controlling for the mediators, the independent explanatory power of the predictor remained. The total effect (β = 2.261, 95% CI 1.752–2.770) reflected the overall strength of the association between targeted online health information and TMDs-5T scores. Furthermore, after controlling age, sex and education level, the indirect effects of anxiety (β = 0.096, 95% CI 0.031–0.161), cyberchondria (β = 0.026, 95% CI 0.003–0.049) and somatization (β = 0.209, 95% CI 0.159–0.258) serve not only as partial mediators in the relationship between targeted online health information and the severity of TMDs but also form a sequential chain mediation.

**Table 5.** Chain mediation model between targeted online health information and the scores of TMDs with SSS-8, GAD-7 and CSS-12.

Effect	Path	Standardized β	Standard error	*p*-value	95% CI
Direct effect	Targeted online health information → TMDs-5T	1.003	0.236	<0.001*	0.538–1.467
Indirect effect					
	Targeted online health information → GAD-7	0.271	0.299	0.364	−0.316–0.858
	Targeted online health information → CSS-12	3.940	0.812	<0.001*	2.346–5.534
	Targeted online health information → SSS-8	4.089	0.490	<0.001*	3.127–5.050
	SSS-8 → GAD-7	0.534	0.024	<0.001*	0.488–0.581
	SSS-8 → CSS-12	0.186	0.088	0.035*	0.013–0.360
	GAD-7 → CSS-12	0.702	0.112	<0.001*	0.481–0.922
	GAD-7 → TMDs-5T	0.096	0.033	0.004*	0.031–0.161
	CSS-12 → TMDs-5T	0.026	0.012	0.027*	0.003–0.049
	SSS-8 → TMDs-5T	0.209	0.025	<0.001*	0.159–0.258
	Targeted online health information → SSS-8 → GAD-7 → TMDs-5T	0.210	0.094	0.026*	0.026–0.395
	Targeted online health information → SSS-8 → CSS-12 → TMDs-5T	0.020	0.015	0.186	−0.010–0.050
	Targeted online health information → GAD-7 → CSS-12 → TMDs-5T	0.005	0.006	0.423	−0.007–0.017
Total effect	Targeted online health information → TMDs-5T	2.261	0.259	<0.001*	1.752–2.770

*: indicates significant differences between groups (p < 0.05) GAD: generalised anxiety disorder; SSS: somatic symptom scale; CSS: cyberchondria severity scale; TMDs-5T: five temporomandibular disorders symptoms; CI: confidence interval.

**Fig. 2.** Association of Targeted online health information (X) and the scores of TMDs-5T (Y) mediated by GAD-7, CSS-12 and SSS-8. GAD: generalised anxiety disorder; SSS: somatic symptom scale; CSS: cyberchondria severity scale; TMDs-5T: five temporomandibular disorders symptoms. *p value < 0.05, **p value < 0.01, ***p value < 0.001.

## 4. Discussion

This study systematically investigates the significant association between targeted online health information and the severity of TMDs, and to further identify anxiety, cyberchondria and somatic symptoms as key components in a serial mediation pathway. As a result, the null hypothesis was rejected.

Our study revealed that targeted online health information delivery, anxiety, somatization and cyberchondria were all significantly and positively associated with TMDs-5T scores. These findings align with previous research, underscoring the role of psychological factors in TMD pathogenesis [35, 36]. TMDs are recognized as typical psychosomatic conditions, in which the development and progression of symptoms are strongly influenced by individual psychological states, cognitive appraisals and behavioral responses [37, 38, 39]. Our results further support this notion by showing that the total effect of targeted online health information delivery on TMD severity was statistically significant.

The mediation analysis identified both independent and chain mediation effects. Specifically, the direct effect of targeted online health information on TMDs scores remained statistically significant, underscoring its unique contribution beyond psychological variables. Previous research has indicated that, although online health information may temporarily alleviate uncertainty, the vast volume of health content available on the Internet is often characterized by significant inaccuracy [40, 41, 42]. It is important to distinguish between reliable and low-quality sources, as the observed psychological effects are likely driven by unverified or misleading content, while information from professional sources may support informed self-management and pose minimal risk. This distinction has practical implications for patient education and clinical guidance. Prolonged and repeated exposure to such unverified information could intensify illness preoccupation and misinterpretation of symptoms, which may, in turn, lead to heightened anxiety and potentially trigger compulsive self-monitoring behaviors. These behaviors might involve sustained, repetitive, and low-intensity teeth clenching or frequent mouth opening and closing, which could consequently contribute to worsening TMD-related pain. The indirect effect further indicated stepwise pathway, in which somatic symptoms were positively associated with heightened anxiety, which subsequently led to increased cyberchondria, thereby creating a cumulative psychological pathway that amplified the influence of targeted online health content on TMDs burden. Importantly, even after adjusting for these psychological variables, targeted online health information remains a significant independent predictor of the severity of TMDs, which highlights its distinctive explanatory value beyond psychological comorbidities. With the rapid expansion of online medical information sources, a growing number of individuals are seeking health-related information through the internet [43]. By leveraging big data analytics and personalized recommendation algorithms, digital platforms can now deliver health content with high precision, based on users’ search histories, browsing patterns and demographic characteristics, thereby enhancing both the relevance and accessibility of medical information. However, in the absence of appropriate safeguards to detect psychological vulnerability, exposure to large volumes of low-quality or scientifically inaccurate information may result in unintended psychological consequences, particularly among individuals with heightened sensitivity. These consequences may include information-induced anxiety, compulsive reassurance-seeking behaviors and symptom amplification, all of which can further increase somatic vigilance and contribute to distorted health perceptions [40, 44]. Notably, a substantially higher proportion of individuals with severe TMDs perceived the impact of online health information as negative. This observation suggests that excessive exposure to health-related content may not always be beneficial and may, in fact, trigger maladaptive cognitive responses.

It is worth emphasizing that, in contrast to previous studies that primarily focused on bivariate relationships between anxiety, somatic symptoms or cyberchondria and TMDs, the present study integrates the influence of increasingly prevalent digital health technologies into the analytical framework. This inclusion not only captures a contemporary and increasingly relevant aspect of the digital health landscape but also enables a more integrated understanding of the mechanisms linking psychological distress with symptom exacerbation in TMDs. By identifying targeted online health information as an upstream factor, this study examines associations that connects digital exposure to psychological responses and clinical outcomes. Inspection of the standardized β coefficients from the mediation analysis indicates that somatic symptoms (SSS-8, β = 0.209) and anxiety (GAD-7, β = 0.096) exert stronger effects on TMD severity than cyberchondria (CSS-12, β = 0.026), suggesting that cyberchondria functions as a key intermediary node rather than the primary predictor. This model enhances our understanding of how online health content may related to a sequence of psychological responses. From the perspective of the biopsychosocial model, the impact of digital health information can be understood mainly through psychosocial pathways. Anxiety, somatization and cyberchondria may amplify symptom perception, while the easy access to online information and pressure for self-managed care may reinforce maladaptive behaviors such as reassurance seeking. Rather than existing outside traditional aetiology, digital exposure may function as a modern psychosocial factor that contributes to the chronic progression of TMDs. Beginning with anxiety, cyberchondria and somatization, and potential linked to greater symptom severity of TMDs. The study addresses a critical gap in the literature and provides novel insight into the “information exposure—psychological stress—symptom perception” cascade within the digital healthcare environment.

Beyond its clinical implications, this study raises important ethical and regulatory considerations in the era of algorithm-driven health communication. Clinically, dentists and primary care professionals can identify patients at risk of information-induced symptom exacerbation through brief pre-consultation communication: asking about the frequency of online health information searches, symptom concerns, or repeated self-monitoring behaviors, and observing heightened anxiety or somatic sensitivity. Using a questionnaire similar to that employed in this study may further help characterize patients. Early identification allows for education on reliable information sources and referral to psychological support when needed. Technology companies should be held to higher standards of algorithmic ethics, including the implementation of psychological risk filters and responsible recommendation protocols. Policymakers are encouraged to develop regulatory frameworks that govern digital health information exposure, particularly for platforms delivering semi-medical content. At the individual level, users should be encouraged to limit personalized recommendation functions, reduce the frequency of unverified health information consumption, and instead seek medical advice from licensed and trustworthy professionals. Collectively, these measures could help mitigate the unintended adverse consequences of digital health content and promote a safer, more supportive online health environment for individuals with TMDs and other psychosomatic conditions.

This study has limitations. First, its cross-sectional design precludes causal inference; the sequence and direction of the chain mediation require verification through longitudinal data. Additionally, psychological variables were assessed via self-report measures. While these scales have good reliability and validity, they may not fully capture participants’ psychological states. Also, the TMDs-5T is a self-reported diagnostic tool; although validated and reliable, it may be affected by recall bias and cannot fully substitute for a clinical examination. Future studies could combine questionnaire screening with professional clinical assessment to improve diagnostic accuracy. Future studies could complement self-reports with clinical interviews or physiological markers to enhance assessment accuracy. Secondly, the study sample was primarily drawn from the community with overall mild TMDs severity, potentially limiting generalizability. Future research should expand to clinical populations or participants with chronic severe conditions. Thirdly, psychological variables were assessed via self-report scales, which despite good reliability and validity, could benefit from supplementation with interviews or physiological measures to improve accuracy. Finally, the model did not include other potential confounding factors such as life stress, comorbid psychosomatic disorders (*e.g.*, fibromyalgia), or socio-economic variables, which may have led to some overestimation of the associations between targeted online health information and TMD severity. Future research should incorporate these factors to provide a more comprehensive understanding of TMD determinants.

## 5. Conclusions

Exposure to targeted online health information is associated with greater severity of TMDs, and that this association is sequentially mediated by anxiety, cyberchondria, and somatization. These findings highlight the urgent need for algorithmic ethics, policy regulation, and user awareness to protect vulnerable individuals in the digital health environment.
